# Discrimination Experiences Among Medical Students

**DOI:** 10.1001/jamanetworkopen.2025.37871

**Published:** 2025-10-16

**Authors:** Mytien Nguyen, Shruthi Venkataraman, Gabriel Abrams, Karina Pereira-Lima, Tonya Fancher, Amy N. Addams, Christopher J. Moreland, Dowin H. Boatright, Lisa M. Meeks

**Affiliations:** 1 Department of Immunobiology, Yale School of Medicine, New Haven, Connecticut; 2 Department of Emergency Medicine, New York University Grossman School of Medicine, New York; 3 University of Michigan Medical School, Ann Arbor; 4 Department of Anesthesiology, University of Michigan Medical School, Ann Arbor; 5 Division of General Internal Medicine, University of California, Davis, School of Medicine, Sacramento; 6 Association of American Medical Colleges, Washington, DC; 7 Department of Internal Medicine, Dell Medical School at the University of Texas at Austin, Austin; 8 Department of Medical Education, University of Illinois College of Medicine, Chicago

## Abstract

**Importance:**

Previous research has documented the prevalence of discrimination based on race, ethnicity, sex, and sexual orientation among medical students; however, discrimination experienced by medical students with disabilities, and the impact of intersecting identities on these experiences, remains underexplored.

**Objective:**

To examine the association of general, gender-based, and race-based discrimination with disability status, sex, race, and ethnicity during medical school.

**Design, Settings, and Participants:**

This cross-sectional study surveyed graduating medical students in US accredited MD-granting medical schools from 2020 to 2022. Analyses were conducted from October to November 2024.

**Main Outcomes and Measures:**

Modified Poisson regression was used to estimate the relative risk of disability status, sex, race, and ethnicity with self-reported experiences of general, gender-based, and race-based discrimination and the intersection of disability status, sex, race, and ethnicity.

**Results:**

Among 45 705 graduating medical students, 3863 (8.5%) reported having a disability; 24 163 (52.9%) identified as female; and 10 100 (22.1%) identified as Asian, 2661 (5.8%) as Black, 4524 (9.9%) as Hispanic, 25 154 (55.0%) as White, and 3266 (7.1%) as other race or ethnicity. Medical students with disability (MSWD) were more likely than their peers without disability to report general discrimination (relative risk [RR], 1.57; 95% CI, 1.50-1.65), gender-based discrimination (RR, 1.64; 95% CI, 1.57-1.72), race-based discrimination (RR, 1.55; 95% CI, 1.44-1.67), and multiple types of discrimination (RR, 1.82; 95% CI, 1.71-1.91). Asian, Black, and Hispanic female MSWD were more likely to report general and race-based discrimination than their White male peers without disability, with the prevalence of general, gender-based, and race-based discrimination among female Asian, Black, and Hispanic MSWD being greater than 25%. Compared with White male students without disability, Asian and Black female MSWD reported the highest risk of general discrimination (Asian: RR, 2.40; 95% CI, 2.09-2.77; Black: RR, 2.58; 95% CI, 2.17-3.06). White and Asian female MSWD reported the highest risk of gender-based discrimination (White: RR, 4.65; 95% CI, 4.30-5.04; Asian: RR, 4.41; 95% CI, 3.85-5.05). Asian and Black female MSWD reported the highest risk of race-based discrimination (Asian: RR, 8.53; 95% CI, 7.26-10.01; Black: RR, 12.48; 95% CI, 10.76-14.47). Finally, Asian and Black female MSWD reported the highest risk of multiple types of discrimination (Asian: RR, 6.50; 95% CI, 5.63-7.52; Black: RR, 7.21; 95% CI, 6.10-8.52).

**Conclusions:**

In this cross-sectional study of US medical students, Asian, Black, and Hispanic female MSWD were more likely to report general and race-based discrimination, whereas White and Asian female MSWD were more likely to report gender-based discrimination during medical school. These findings highlight the need to address intersecting forms of discrimination for medical students with disabilities.

## Key Points

**Question:**

What is the association of general, gender-based, and race-based discrimination with students’ disability status, sex, race, and ethnicity during medical school?

**Findings:**

Among 45 705 graduating medical students in this cross-sectional study, Asian, Black, and Hispanic female students with disability were more likely to report general and race-based discrimination than White male students without disability. White and Asian female students with disability were more likely to report gender-based discrimination than White male students without disability.

**Meaning:**

These findings suggest that students with disabilities with intersecting identities experience different types of discrimination based on race, ethnicity, and sex, emphasizing the importance of addressing multiple, overlapping forms of discrimination in medical education.

## Teaser

This cross-sectional study examines the association of general, gender-based, and race-based discrimination with disability status, sex, and race and ethnicity among medical students in the US.

## Introduction

Discrimination in undergraduate medical education has been linked to depression and impaired professional and personal identity formation.^1,2,3,4,5^ Medical students who face discrimination are significantly more likely to disengage from their studies,^2,3^ with some ultimately leaving medical school.^3,6^ In recent years, the demographic composition of medical schools has shifted, with an increasing number of medical students identifying as disabled.^7,8^ Among medical students, attention deficit/hyperactivity disorder, learning disabilities, and psychological disabilities are the most commonly reported.^7^ Like other underrepresented groups, medical students with disability (MSWD) are vulnerable to discrimination, which may manifest as explicit biases, microaggressions, and structural barriers that hinder their academic performance, personal well-being, and long-term career aspirations.^9,10,11,12,13^ The impact of discrimination on MSWD can be further compounded when intersecting identities, such as race, ethnicity, and sex, are considered. Medical students who belong to multiple marginalized groups often experience unique forms of discrimination and structural inequities, which can exacerbate the challenges they encounter in the learning environment.^3,14,15^

While substantial research has documented the prevalence of discrimination based on race, ethnicity, gender, and sexual orientation among medical students,^1,2,3,4,5,6,16,17,18^ discrimination experienced by MSWD remains underexplored. Even less attention is directed to medical students who navigate intersecting identities, such as disability alongside marginalized sex and racial groups. This lack of understanding of how intersectionality shapes medical students’ educational experiences presents a critical gap in the literature. This study aims to fill this gap by exploring the prevalence of general, gender-based, and race-based discrimination across the intersections of disability status, sex, race, and ethnicity.

## Methods

We conducted a cross-sectional study of medical students enrolled in MD-granting medical schools who completed the Graduation Questionnaire (GQ) administered by the Association of American Medical Colleges (AAMC) between 2020 and 2022. This study was deemed exempt from review by the University of Michigan Institutional Review Board because the data were deidentified. This study followed the Strengthening the Reporting of Observational Studies in Epidemiology (STROBE) reporting guidelines.

Demographic variables included self-reported disability status, race, ethnicity, and sex. Disability is self-reported on the GQ and includes the following options: attention deficit/hyperactivity disorder, chronic health disability, deaf or hard of hearing, learning disability, mobility disability, psychological disability, and visual disability. Self-reported race and ethnicity were surveyed on the American Medical College Application Service (AMCAS), including more than 20 racial and ethnic options that were recategorized into 5 distinct categories: Asian, Black, Hispanic, White, and Other. Students who reported Hispanic were categorized as Hispanic regardless of racial category. Other students include students who reported American Indian, Alaska Native, Hawaiian Native, or Pacific Islander; those who identified as “other” or more than 1 racial identity; and students who had unknown racial and ethnic identity.

The GQ includes questions about discrimination across multiple domains: general discrimination, gender-based discrimination, and race-based discrimination. General discrimination includes public belittlement or humiliation, being threatened with physical harm, or being physically harmed. Gender-based discrimination includes receiving lower evaluations or grades solely because of gender rather than performance, being denied opportunities for training or rewards based on gender, being subjected to offensive sexist remarks or names, being subjected to unwanted sexual advances, or being asked to exchange sexual favors for grades or other rewards. Race-based discrimination includes receiving lower evaluations or grades solely because of race rather than performance, being denied opportunities for training or rewards on the basis of race, or being subjected to offensive racist remarks or names. Experiences of discrimination were determined for students who reported 1 or more instances of discrimination. Experiences of multiple discrimination types were defined as students who reported 2 or more types of discrimination (eg, both gender- and race-based discrimination).

### Statistical Analysis

Analyses were conducted from October 2024 to November 2024. We used descriptive statistics to assess the prevalence of general, gender-based, and race-based discrimination by disability identity, sex, race, and ethnicity. Modified Poisson regression was used to estimate the relative risk of disability status, sex, and race and ethnicity with experiences of discrimination, and the intersection of disability status, sex, and race and ethnicity. Statistical significance was determined with a 2-sided *P* value of <.05. Analyses were performed using Stata, version 18.0 (StataCorp LLC).

## Results

Among 45 705 graduating medical students, 3863 (8.5%) reported having a disability. A total of 24 163 (52.9%) students identified as female and 21 542 (47.1%) as male, and 10 100 (22.1%) identified as Asian, 2661 (5.8%) as Black, 4524 (9.9%) as Hispanic, 25 154 (55.0%) as White, and 3266 (7.1%) as other race and ethnicity (Table 1).

**Table 1.** Characteristics of the Study Cohort

Characteristic	Medical students, No. (%) (N = 45 705)
Disability status	
No	41 842 (91.5)
Yes	3863 (8.5)
Sex	
Male	21 542 (47.1)
Female	24 163 (52.9)
Race and ethnicity	
Asian	10 100 (22.1)
Black	2661 (5.8)
Hispanic	4524 (9.9)
Other^a^	3266 (7.1)
White	25 154 (55.0)
Intersectional identity	
Male, Asian, without disability	4299 (9.4)
Male, Black, without disability	860 (1.9)
Male, Hispanic, without disability	1887 (4.1)
Male, Other, without disability	1350 (3.0)
Male, White, without disability	11 414 (25.0)
Male, Asian, with disability	199 (0.4)
Male, Black, with disability	84 (0.2)
Male, Hispanic, with disability	255 (0.6)
Male, Other, with disability	156 (0.3)
Male, White, with disability	1038 (2.3)
Female, Asian, without disability	5316 (11.6)
Female, Black, without disability	1553 (3.4)
Female, Hispanic, without disability	2095 (4.6)
Female, White, without disability	11 510 (25.2)
Female, Other, without disability	1558 (3.4)
Female, Asian, with disability	286 (0.6)
Female, Black, with disability	164 (0.4)
Female, Hispanic, with disability	287 (0.6)
Female, Other, with disability	202 (0.4)
Female, White, with disability	1192 (2.6)

^a^ Other race and ethnicity included students who reported American Indian, Alaska Native, Hawaiian Native, or Pacific Islander; those who identified as “other” or more than 1 racial identity; and students who had unknown racial and ethnic identity.

### General Discrimination

Among 45 705 students in the cohort, 10 138 (22.2%) reported general discrimination (Table 2). MSWD reported a higher risk of general discrimination compared with their peers without disability (relative risk [RR], 1.57; 95% CI, 1.50-1.65). Females were more likely to report general discrimination than males (5758 of 24 163 [23.8%] vs 4380 of 21 542 [20.3%]; RR, 1.17; 95% CI, 1.13-1.21). Across racial and ethnic groups, Black students reported the highest rates of general discrimination (767 of 2661 [28.8%]), with an RR of 1.41 (95% CI, 1.32-1.50) compared with White students. Hispanic and Asian students also reported higher risks of general discrimination than White students (Hispanic: RR, 1.18; 95% CI, 1.12-1.26; Asian: RR, 1.12; 95% CI, 1.07-1.17).

**Table 2.** Association of Sociodemographic Identity With General Discrimination

Variable	Total, No.	General discrimination, No. (%)	RR (95% CI)
Total	45 705	10 138 (22.2)	NA
Disability status			
No	41 842	8849 (21.1)	1 [Reference]
Yes	3863	1289 (33.4)	1.57 (1.50-1.65)
Sex			
Male	21 542	4380 (20.3)	1 [Reference]
Female	24 163	5758 (23.8)	1.17 (1.13-1.21)
Race and ethnicity			
Asian	10 100	2312 (22.8)	1.12 (1.07-1.17)
Black	2661	767 (28.8)	1.41 (1.32-1.50)
Hispanic	4524	1099 (24.2)	1.18 (1.12-1.26)
Other^a^	3266	824 (25.2)	1.23 (1.16-1.32)
White	25 154	5136 (20.4)	1 [Reference]
Intersectional identity			
Male, Asian, without disability	4299	938 (21.8)	1.23 (1.14-1.31)
Male, Black, without disability	860	203 (23.6)	1.33 (1.17-1.51)
Male, Hispanic, without disability	1887	385 (20.4)	1.15 (1.04-1.26)
Male, Other, without disability	1350	312 (23.1)	1.30 (1.17-1.44)
Male, White, without disability	11 414	2022 (17.7)	1 [Reference]
Male, Asian, with disability	199	71 (35.7)	2.01 (1.66-2.43)
Male, Black, with disability	84	18 (21.4)	1.20 (0.80-1.82)
Male, Hispanic, with disability	255	86 (33.7)	1.90 (1.59-2.27)
Male, Other, with disability	156	58 (37.1)	2.09 (1.70-2.58)
Male, White, with disability	1038	287 (27.6)	1.56 (1.40-1.73)
Female, Asian, without disability	5316	1181 (22.2)	1.25 (1.17-1.33)
Female, Black, without disability	1553	471 (30.3)	1.71 (1.57-1.86)
Female, Hispanic, without disability	2095	525 (25.1)	1.41 (1.30-1.53)
Female, Other, without disability	1558	372 (23.8)	1.34 (1.22-1.48)
Female, White, without disability	11 510	2440 (21.1)	1.19 (1.13-1.26)
Female, Asian, with disability	286	122 (42.6)	2.40 (2.09-2.77)
Female, Black, with disability	164	75 (45.7)	2.58 (2.17-3.06)
Female, Hispanic, with disability	287	103 (35.8)	2.02 (1.72-2.37)
Female, Other, with disability	202	82 (40.5)	2.29 (1.93-2.72)
Female, White, with disability	1192	387 (32.4)	1.83 (1.67-2.00)

^a^ Other race and ethnicity included students who reported American Indian, Alaska Native, Hawaiian Native, or Pacific Islander; those who identified as “other” or more than 1 racial identity; and students who had unknown racial and ethnic identity.

Intersectional analyses revealed notable differences across groups. Among males, Asian and Hispanic MSWD were more likely to experience general discrimination than White male students without disability. Specifically, 71 of 199 Asian male MSWD (35.7%; RR, 2.01; 95% CI, 1.66-2.43) and 86 of 255 Hispanic male MSWD (33.7%; RR, 1.90; 95% CI, 1.59-2.27) reported experiencing general discrimination. Among females, Black MSWD were more likely to experience general discrimination, with 75 of 164 (45.7%) reporting general discrimination (RR, 2.58; 95% CI, 2.17-3.06). Asian female MSWD were also more likely (122 of 286 [42.6%]) to report general discrimination (RR, 2.40; 95% CI, 2.09-2.77) compared with White students without disability.

### Gender-Based Discrimination

Table 3 demonstrates the gender-based discrimination findings, reported by 9990 students (21.9%) in the cohort. Female students were significantly more likely to report gender-based discrimination than male students (7601 of 24 163 [31.5%] vs 2389 of 21 542 [11.1%]; RR, 2.83; 95% CI, 2.71-2.95). MSWD were disproportionately impacted, with a significantly higher risk of gender-based discrimination than nondisabled students (1319 of 3863 [34.1%] vs 8671 of 41 842 [20.7%]; RR, 1.64; 95% CI, 1.57-1.72). While White and Hispanic students reported similar rates of gender-based discrimination, Asian and Black students reported slightly lower rates of gender-based discrimination (Asian: RR, 0.91; 95% CI, 0.86-0.95; Black: RR, 0.93; 95% CI, 0.86-1.01).

**Table 3.** Association of Sociodemographic Identity With Gender-Based Discrimination

Variable	Total, No.	Gender-based discrimination, No. (%)	RR (95% CI)
Total	45 705	9990 (21.9)	NA
Disability status			
No	41 842	8671 (20.7)	1 [Reference]
Yes	3863	1319 (34.1)	1.64 (1.57-1.72)
Sex			
Male	21 542	2389 (11.1)	1 [Reference]
Female	24 163	7601 (31.5)	2.83 (2.71-2.95)
Race and ethnicity			
Asian	10 100	2040 (20.1)	0.91 (0.86-0.95)
Black	2661	556 (20.8)	0.93 (0.86-1.01)
Hispanic	4524	1004 (22.1)	1.00 (0.94-1.06)
Other^a^	3266	798 (24.4)	1.10 (1.03-1.17)
White	25 154	5592 (22.2)	1 [Reference]
Intersectional identity			
Male, Asian, without disability	4299	424 (9.8)	0.94 (0.84-1.04)
Male, Black, without disability	860	80 (9.3)	0.88 (0.71-1.10)
Male, Hispanic, without disability	1887	201 (10.6)	1.01 (0.88-1.17)
Male, Other, without disability	1350	171 (12.6)	1.21 (1.04-1.40)
Male, White, without disability	11 414	1194 (10.4)	1 [Reference]
Male, Asian, with disability	199	41 (20.6)	1.96 (1.49-2.60)
Male, Black, with disability	84	19 (22.6)	2.16 (1.45-3.22)
Male, Hispanic, with disability	255	49 (19.2)	1.83 (1.42-2.37)
Male, Other, with disability	156	41 (26.2)	2.51 (1.92-3.28)
Male, White, with disability	1038	169 (16.2)	1.55 (1.34-1.80)
Female, Asian, without disability	5316	1443 (27.1)	2.59 (2.42-2.78)
Female, Black, without disability	1553	387 (24.9)	2.38 (2.15-2.63)
Female, Hispanic, without disability	2095	636 (30.3)	2.90 (2.66-3.15)
Female, Other, without disability	1558	487 (31.2)	2.98 (2.72-3.27)
Female, White, without disability	11 510	3648 (31.6)	3.02 (2.85-3.21)
Female, Asian, with disability	286	132 (46.1)	4.41 (3.85-5.05)
Female, Black, with disability	164	70 (42.6)	4.08 (3.39-4.91)
Female, Hispanic, with disability	287	118 (41.1)	3.93 (3.38-4.55)
Female, Other, with disability	202	99 (49)	4.68 (4.03-5.44)
Female, White, with disability	1192	581 (48.7)	4.65 (4.30-5.04)

^a^ Other race and ethnicity included students who reported American Indian, Alaska Native, Hawaiian Native, or Pacific Islander; those who identified as “other” or more than 1 racial identity; and students who had unknown racial and ethnic identity.

When examined through an intersectional lens, striking disparities were revealed. Nearly half (581 of 1192 [48.7%]) of White female MSWD reported gender-based discrimination, compared with 1194 of 11 414 (10.4%) White male students without disability (RR, 4.65; 95% CI, 4.30-5.04). Asian female MSWD faced similarly high rates, with 132 of 286 (46.1%) reporting gender-based discrimination (RR, 4.41; 95% CI, 3.85-5.05). Among males, 19 of 84 Black male MSWD (22.6%) and 41 of 199 Asian male MSWD (20.6%) reported gender-based discrimination compared with 1194 of 11 414 White male students without disability (10.4%) (Black: RR, 2.16; 95% CI, 1.45-3.22; Asian: RR, 1.96; 95% CI, 1.49-2.60).

### Race-Based Discrimination

Table 4 highlights that 5409 of 45 705 participants (11.8%) reported race-based discrimination, with significant disparities across demographic groups. MSWD were more likely to report race-based discrimination than their peers without disability (680 of 3863 [17.6%] vs 4729 of 41 842 [11.3%]; RR, 1.55; 95% CI, 1.44-1.67). Female students also were more likely to report race-based discrimination than male students (3181 of 24 163 [13.2%] vs 2228 of 21 542 [10.3%]; RR, 1.27; 95% CI, 1.21-1.33). Black students were the most impacted, with 928 of 2661 [34.8%] reporting race-based discrimination—significantly more than the risk for White students (RR, 8.55; 95% CI, 7.90-9.26). Asian students also faced substantial risk compared with White students, with 2163 of 10 100 [21.4%] reporting race-based discrimination (RR, 5.26; 95% CI, 4.90-5.64). Hispanic students were also more likely to report race-based discrimination than White students, with 750 of 4524 (16.6%) affected (RR, 4.06; 95% CI, 3.72-4.44).

**Table 4.** Association of Sociodemographic Identity With Race-Based Discrimination

Variable	Total, No.	Race-based discrimination, No. (%)	RR (95% CI)
Total	45 705	5409 (11.8)	NA
Disability status			
No	41 842	4729 (11.3)	1 [Reference]
Yes	3863	680 (17.6)	1.55 (1.44-1.67)
Sex			
Male	21 542	2228 (10.3)	1 [Reference]
Female	24 163	3181 (13.2)	1.27 (1.21-1.33)
Race and ethnicity			
Asian	10 100	2163 (21.4)	5.26 (4.90-5.64)
Black	2661	928 (34.8)	8.55 (7.90-9.26)
Hispanic	4524	750 (16.6)	4.06 (3.72-4.44)
Other^a^	3266	543 (16.6)	4.08 (3.70-4.49)
White	25 154	1025 (4.0)	1 [Reference]
Intersectional identity			
Male, Asian, without disability	4299	754 (17.5)	3.62 (3.26-4.02)
Male, Black, without disability	860	230 (26.7)	5.53 (4.82-6.34)
Male, Hispanic, without disability	1887	254 (13.4)	2.78 (2.41-3.20)
Male, Other, without disability	1350	184 (13.6)	2.81 (2.40-3.29)
Male, White, without disability	11 414	552 (4.8)	1 [Reference]
Male, Asian, with disability	199	46 (23.1)	4.77 (3.66-6.23)
Male, Black, with disability	84	29 (34.5)	7.13 (5.25-9.68)
Male, Hispanic, with disability	255	63 (24.7)	5.10 (4.06-6.42)
Male, Other, with disability	156	37 (23.7)	4.90 (3.65-6.57)
Male, White, with disability	1038	79 (7.6)	1.57 (1.25-1.97)
Female, Asian, without disability	5316	1245 (23.4)	4.84 (4.40-5.32)
Female, Black, without disability	1553	570 (36.7)	7.58 (6.83-8.42)
Female, Hispanic, without disability	2095	355 (16.9)	3.50 (3.09-3.97)
Female, Other, without disability	1558	261 (16.7)	3.46 (3.01-3.97)
Female, White, without disability	11 510	324 (2.8)	0.58 (0.50-0.66)
Female, Asian, with disability	286	118 (41.3)	8.53 (7.26-10.01)
Female, Black, with disability	164	99 (60.4)	12.48 (10.76-14.47)
Female, Hispanic, with disability	287	78 (27.1)	5.61 (4.57-6.90)
Female, Other, with disability	202	61 (30.1)	6.24 (4.98-7.81)
Female, White, with disability	1192	70 (5.8)	1.21 (0.95-1.54)

^a^ Other race and ethnicity included students who reported American Indian, Alaska Native, Hawaiian Native, or Pacific Islander; those who identified as “other” or more than 1 racial identity; and students who had unknown racial and ethnic identity.

Among males, 29 of 84 Black MSWD (34.5%) reported race-based discrimination and were more likely to report race-based discrimination compared with male White students without disability (RR, 7.13; 95% CI, 5.25-9.68). In addition, 63 of 255 Hispanic male MSWD (24.7%) reported race-based discrimination—significantly more than male White students without disability (RR, 5.10; 95% CI, 4.06-6.42). Among females, Black MSWD were the most affected, with 99 of 164 (60.4%) reporting race-based discrimination—also significantly more than male White students without disability (RR, 12.48; 95% CI, 10.76-14.47). Asian female MSWD also reported significantly high rates compared with male White students without disability, with 118 of 286 (41.3%) experiencing race-based discrimination (RR, 8.53; 95% CI, 7.26-10.01).

### Experiences of Multiple Discrimination Types

Table 5 outlines the experiences of discrimination by type, showing that 6735 of those in the total cohort (14.7%) experienced more than 1 type of discrimination. MSWD were significantly more likely to face multiple discrimination types compared with students without disability (969 of 3863 [25.0%] vs 5766 of 41 842 [13.8%]; RR, 1.82; 95% CI, 1.71-1.91). Sex differences were also pronounced, with female students more likely to report multiple discrimination types than male students (4578 of 24 163 [18.9%] vs 2157 of 21 542 [10.0%]; RR, 1.89; 95% CI, 1.80-1.98). Across racial and ethnic groups, compared with White students, Black students reported the highest rate of multiple discrimination types (685 of 2661 [25.7%]; RR, 2.35; 95% CI, 2.18-2.53), followed by Asian students (1911 of 10 100 [18.9%]; RR, 1.72; 95% CI, 1.63-1.82) and Hispanic students (785 of 4524 [17.3%]; RR, 1.58; 95% CI, 1.47-1.70).

**Table 5.** Association of Sociodemographic Identity With Experiences of Multiple Discrimination Types

Variable	Total, No.	≥1 Discrimination type, No. (%)	RR (95% CI)
Total	45 705	6735 (14.7)	NA
Disability status			
No	41 842	5766 (13.8)	1 [Reference]
Yes	3863	969 (25.0)	1.82 (1.71-1.91)
Sex			
Male	21 542	2157 (10.0)	1 [Reference]
Female	24 163	4578 (18.9)	1.89 (1.80-1.98)
Race and ethnicity			
Asian	10 100	1911 (18.9)	1.72 (1.63-1.82)
Black	2661	685 (25.7)	2.35 (2.18-2.53)
Hispanic	4524	785 (17.3)	1.58 (1.47-1.70)
Other^a^	3266	601 (18.4)	1.68 (1.55-1.82)
White	25 154	2753 (10.9)	1 [Reference]
Intersectional identity			
Male, Asian, without disability	4299	556 (12.9)	1.86 (1.68-2.06)
Male, Black, without disability	860	149 (17.3)	2.50 (2.12-2.93)
Male, Hispanic, without disability	1887	206 (10.9)	1.57 (1.36-1.82)
Male, Other, without disability	1350	171 (12.6)	1.82 (1.56-2.13)
Male, White, without disability	11 414	791 (6.9)	1 [Reference]
Male, Asian, with disability	199	45 (22.6)	3.26 (2.50-4.25)
Male, Black, with disability	84	19 (22.6)	3.26 (2.18-4.87)
Male, Hispanic, with disability	255	55 (21.5)	3.11 (2.43-3.97)
Male, Other, with disability	156	37 (23.7)	3.42 (2.56-4.57)
Male, White, with disability	1038	128 (12.3)	1.77 (1.49-2.12)
Female, Asian, without disability	5316	1181 (22.2)	3.20 (2.94-3.48)
Female, Black, without disability	1553	435 (28)	4.04 (3.64-4.48)
Female, Hispanic, without disability	2095	432 (20.6)	2.97 (2.67-3.31)
Female, Other, without disability	1558	312 (20)	2.88 (2.56-3.25)
Female, White, without disability	11 510	1533 (13.3)	1.92 (1.77-2.08)
Female, Asian, with disability	286	129 (45.1)	6.50 (5.63-7.52)
Female, Black, with disability	164	82 (50.0)	7.21 (6.10-8.52)
Female, Hispanic, with disability	287	92 (32.1)	4.62 (3.85-5.54)
Female, Other, with disability	202	81 (40.1)	5.78 (4.82-6.93)
Female, White, with disability	1192	301 (25.2)	3.64 (3.23-4.10)

^a^ Other race and ethnicity included students who reported American Indian, Alaska Native, Hawaiian Native, or Pacific Islander; those who identified as “other” or more than 1 racial identity; and students who had unknown racial and ethnic identity.

Intersectional analyses revealed high variability in experiences of multiple discrimination types. Among males, Black and Asian MSWD reported the highest rates, with 22.6% in each group (45 of 199 Asian and 19 of 84 Black) experiencing multiple forms of discrimination—both significantly more than the risk for male White students without disability (Black: RR, 3.26; 95% CI, 2.18-4.87; Asian: RR, 3.26; 95% CI, 2.50-4.25). Among females, Black MSWD were the most impacted, with 82 of 164 (50.0%) reporting multiple types of discrimination—a significantly greater risk compared with male White students without disability (RR, 7.21; 95% CI, 6.10-8.52). Female Asian MSWD also experienced significantly higher prevalence of multiple discrimination types compared with male White students without disability, with 129 of 286 (45.1%) reporting multiple discrimination types (RR, 6.50; 95% CI, 5.63-7.52).

## Discussion

This study of a national cohort of medical students reveals the critical extent of discrimination faced by MSWD, particularly MSWD who are female and of races other than White, who report the highest rate of general, gender-based, and race-based discrimination. More than 40% of female Asian and Black students with disability reported experiencing multiple types of discrimination during medical training.

Although racism in medical education is well documented,^1,4,6,17,19,20^ ableism—defined as prejudice and discrimination against individuals with disabilities^21^—has not received the same attention. Deeply embedded in the culture of medical education, ableism is perpetuated by traditions that emphasize physical and cognitive rigor,^22^ often reinforcing the misconception that having disabilities is incompatible with the demands of clinical practice.^22,23^ These ableist biases can manifest in subtle and covert forms of discrimination, creating a more challenging and exclusionary learning environment for MSWD, underscoring the widespread impact of ableism within the profession.^24,25^ In this study, MSWD were found to have a 57% higher likelihood of reporting general discrimination compared with medical students without disability. This increased likelihood of reports of discrimination may reflect disability stigma in the medical profession that is fueled by misconceptions about the capabilities of individuals with disabilities in clinical settings.^22^ For medical students, disability bias has far-reaching consequences, including exclusion from critical opportunities that could advance their professional development, such as lower match rates and likelihood of induction into honor societies.^13,26^

The impact of discrimination was most pronounced at the intersections of marginalized identities. This study found that female MSWD of races other than White face compounded discrimination, likely due to the collective impact of ableism, racism, and sexism.^4,9^ Black female MSWD were 7 times more likely to report multiple discrimination types compared with their White male peers without disability. This highlights the urgent need for policy interventions and support systems that address the unique challenges faced by multiply marginalized MSWD.^2,3^

Discrimination based on disability, race, gender, or other identities stands in direct opposition to the core values of the medical profession, including equity, compassion, and professionalism.^27^ Physicians and medical educators are entrusted with upholding these standards, and discriminatory behavior—particularly within training environments—not only undermines the well-being of learners as future members of the medical workforce^28^ but also may erode the integrity of the profession and the quality of care patients receive.

Inadequate accommodations are one form of disability discrimination^10,29,30^ and may further exacerbate these challenges as many medical schools fail to provide appropriate or timely accommodations.^10,22,31,32,33,34^ Effective accommodations may improve medical student well-being and reduce burnout,^11,32,35^ yet critical aspects of medical education—such as clinical rotations and standardized patient examinations—frequently lack accessibility.^10,33^ Furthermore, the emphasis on long hours and physical stamina in clinical clerkships can disproportionately affect medical students who require accommodations for chronic illnesses or mobility or sensory disabilities.^33^ The failure to accommodate disabilities actively disadvantages MSWD by tolerating environments that cater to students without disabilities. By neglecting to provide necessary accommodations, medical institutions may contribute to a discriminatory system that limits opportunities, perpetuates marginalization, and undermines diversity in the health care profession.

To create an inclusive training environment, disability accommodations must be precise and designed to address disability-related needs and also incorporate cultural and gender-specific considerations, such as those related to pregnancy or childcare.^10,34,36^ Cultural considerations might include values, norms, communication styles, and expectations shaped by individual backgrounds, which may influence how disability is perceived, disclosed, or managed. For example, cultural stigma surrounding disability in Asian communities^37^ may discourage students from seeking formal accommodations, while distrust in institutional systems in Black and Hispanic communities^38^ can further limit access to support. An inclusive approach should account for these dynamics and ensure that accommodation processes are accessible and responsive to the cultural identities and lived experiences of all students.

### Limitations

This study is limited by lack of substantial data specifically on disability-based discrimination experienced by MSWD that allows for intersectional analysis. While the annual AAMC surveys began assessing discrimination based on disability in 2024, these data are not yet sufficiently powered for intersectional studies. In addition, a key limitation is the reliance on self-reported data for both disability status and discrimination experiences, which may be subject to reporting bias, though such perceptions are nonetheless critical as they can strongly influence well-being and educational outcomes. Importantly, the anonymity afforded by AAMC self-report surveys may provide the protection and psychological safety that MSWD require to disclose sensitive information—potentially resulting in a more accurate accounting of these experiences. Furthermore, experiences of discrimination may vary by disability type. Prior research has shown that students with multiple disabilities are more likely to report burnout compared to their peers.^28^ Future studies should investigate the nature of discrimination experienced by students with disability, considering both specific disability types and the intersections of multiple disabilities.

## Conclusions

In this cross-sectional study of graduating medical students, findings reveal that female students of races other than White with disabilities report the highest rates of discrimination, underscoring the profound impact of intersecting ableism, racism, and sexism in medical education. Addressing this inequity requires medical schools and accrediting bodies to confront all forms of discrimination, adopt intersectional frameworks for disability inclusion, and cultivate learning environments where diversity is valued and medical students at the intersection of multiple marginalized identities—especially female MSWD from minoritized racial and ethnic groups— are empowered to thrive in their journey to becoming physicians.

## Notes

Supplement.
Data Sharing Statement
