# Supplementary material for: Discrimination Experiences Among Medical Students
Source: JAMA Netw Open. 2025 Oct 16;8(10):e2537871. doi: 10.1001/jamanetworkopen.2025.37871 (PMC12531876; doi:10.1001/jamanetworkopen.2025.37871)
Supplement: Supplement. — Data Sharing Statement [file jamanetwopen-e2537871-s001.pdf]

Data Sharing Statement

Nguyen. Discrimination Experiences Among Medical Students. *JAMA Netw Open*. Published October 16, 2025. doi:10.1001/jamanetworkopen.2025.37871

Data
Data available: No
